# The Relationship Between Social Power and Sexual Objectification: Behavioral and ERP Data

**DOI:** 10.3389/fpsyg.2019.00057

**Published:** 2019-01-25

**Authors:** Lijuan Xiao, Baolin Li, Lijun Zheng, Fang Wang

**Affiliations:** ^1^Beijing Key Laboratory of Applied Experimental Psychology, National Demonstration Center for Experimental Psychology Education, Faculty of Psychology, Beijing Normal University, Beijing, China; ^2^Key Laboratory of Cognition and Personality, Faculty of Psychology, Southwest University, Chongqing, China; ^3^School of Psychological and Cognitive Sciences, Peking University, Beijing, China

**Keywords:** power, sexual objectification, analytical processing, configural processing, inversion effect

## Abstract

Sexual objectification is very common in modern Western societies, especially toward women. Previous research has suggested that in Western cultures, social power could lead to objectification. Specifically, power activates an approaching tendency toward useful targets, in turn leading to instrumental objectification and sexual objectification of targets. However, previous research has mostly focused on Western cultures, and the neural correlates underlying this phenomenon remain unclear. To examine whether the effects of power can be generalized to Chinese cultural contexts and how power promotes the objectification of sexualized bodies, we conducted two studies using Chinese samples. In Study 1, we replicated the behavioral effects of social power on sexual objectification. Specifically, we found that power increased sexual objectification toward sexualized female rather than male bodies. In Study 2, we examined the absence of an N170 amplitude inversion effect as a possible neural correlate of sexual objectification and replicated the effects of power on sexual objectification through event-related potentials (ERPs). For participants in a high-power group, the N170 amplitude inversion effect emerged when processing sexualized male bodies (less sexual objectification) but not female bodies (more sexual objectification); this effect was not seen for those participants in a low-power group. Our findings provide behavioral and neural data that power leads to increased sexual objectification toward sexualized women in Chinese participants.

## Introduction

There is currently a trend in Western culture for women to be depicted in an objectified manner in both social interactions and mass media presentations. For example, women report being gazed at by others more than men do during social interactions, and women in visual media are usually depicted with an emphasis on the body and body parts, while men are portrayed with an emphasis on the head and face (Fredrickson and Roberts, 1997). These findings suggest the existence of sexual objectification. Fredrickson and Roberts (1997) proposed that in Western cultures, women in social interactions often encounter sexual objectification, in which a woman’s body or body parts are separated from her and reduced to mere instruments or regarded as if they represent the entire person. Specifically, sexual objectification is a form of appearance focus directed toward a woman’s sexual body or body parts. Sexual objectification is a common occurrence in daily life, arising in various forms of media and interpersonal encounters. It can present as indirect body monitoring or in direct forms, such as rape and sexual harassment (Fredrickson and Roberts, 1997).

Once sexually objectified, women gradually internalize an objectifying observer’s perspective on their own bodies, becoming preoccupied with their own physical appearance, which is called self-objectification (Fredrickson and Roberts, 1997). Self-objectification can in turn lead to physical, mental, and social consequences (Loughnan and Vaes, 2017), such as body shame, social physique anxiety (Calogero, 2004), reduced cognitive performance (Quinn et al., 2006; Gay and Castano, 2010), and eating disorders and depression (Peat and Muehlenkamp, 2011). In the eyes of objectifiers, objectified women have less warmth, competence, and humanity than their non-objectified counterparts (Heflick and Goldenberg, 2009; Loughnan et al., 2010; Heflick et al., 2011; Vaes et al., 2011). Additionally, the objectified share this view and see themselves in the manner in which they are seen by their objectifiers: as lacking warmth, competence, morality, and humanity (Loughnan et al., 2017). Due to its widespread and salient harmful effects, especially on women, sexual objectification has attracted increasing attention in recent years.

From the theoretical framework of sexual objectification, research on cognitive objectification (Bernard et al., 2018b) has suggested that sexualized women (as opposed to sexualized men) are cognitively perceived in the manner of objects, that is, through analytical processing (e.g., Bernard et al., 2012, 2017, 2018b; Gervais et al., 2012). Specifically, Bernard et al. (2012) proposed the sexualized-body inversion hypothesis (SBIH), which suggests that sexual objectification would reduce or eliminate the inversion effect for sexualized women but not for sexualized men. The inversion effect is defined as inverted stimuli being more difficult to recognize than upright ones (Yin, 1969). Scholars doubted the validity of the sexualized-body inversion effect observed by Bernard et al. (2012) and suggested that the physical features of the stimulus matter (e.g., there is more asymmetry in female targets than male targets) (Tarr, 2013; Schmidt and Kistemaker, 2015). However, later research replicated the effects with the same or similar targets used in Bernard et al. (2012) (e.g., Kostic, 2013; Bernard et al., 2015; Civile and Obhi, 2016; Civile et al., 2016), and suggested that the sexualized-body inversion effect cannot be explained by the physical features of the stimulus *per se*, as the sexual connotation of the target’s posture plays a crucial role in cognitive objectification (Bernard et al., 2018c). Evidence from behavioral data suggested that female sexual body parts could be better recognized in isolation, whereas male sexual body parts are better recognized within the context of the entire body (Gervais et al., 2012). Bernard et al. (2015) also found that the occurrence of sexual objectification results from a focus on the sexual body parts of women. More recently, ERP evidence of cognitive objectification was provided by Bernard et al. (2017, 2018a,c). It was found that there is an N170 amplitude inversion effect for inverted (vs. upright) men’s bodies but not for women’s bodies, regardless of the extent of target sexualization (non-sexualized vs. sexualized). N170 is a negative peak approximately 170 ms after stimulus onset at occipitotemporal regions (Thierry et al., 2006), and the N170 component is accepted as a face-selective component (Itier and Taylor, 2004) that might reflect configural information during facial processing (Rossion et al., 2000; Goffaux et al., 2003). These findings suggested that women’s bodies are more likely to be perceived with a localized focus, especially the sexual regions, rather than as entire bodies. That is, sexualized women are processed more analytically (i.e., as sexual objects rather than as people) compared to sexualized men. Indeed, research has suggested that face/body perception is mainly based on configural processing (Reed et al., 2006), while object perception is based on analytical processing (Tanaka and Farah, 1993). Specifically, configural processing is related to the processing of the relationships between the features of a stimulus (Maurer et al., 2002), while analytical processing primarily depends on the processing of each feature individually (Diamond and Carey, 1986; Leder and Bruce, 2000). The inversion effect for faces has been suggested to be an indicator of configural processing (Yin, 1969), and inverted human stimuli are more difficult to recognize than inverted objects (Reed et al., 2003, 2006). The inversion of faces and bodies but not of objects disrupts recognition performance, suggesting that configural processing is mainly involved in face/body perception rather than object perception (Civile and Obhi, 2016).

In addition, there are many relevant social factors that can promote the objectification process, such as social power. As a common phenomenon, power plays an important role in daily social life that can be defined as an individual’s capacity to control resources and influence others, including the ability to administer rewards and punishments (Galinsky et al., 2003; Keltner et al., 2003). Possessing power is accompanied by various cognitive outcomes, such as increased independent self-construals (Lee and Tiedens, 2001), increased action orientation (Galinsky et al., 2003), greater approach behaviors (Keltner et al., 2003), and increased association with instrumentality when approaching social targets (Gruenfeld et al., 2008). Guinote (2017) concluded that power affects people in terms of activating, wanting and goal seeking. The power-approach theory suggests that higher levels of power are associated with increased rewards and freedom, which, in turn, activate power holders’ approach tendencies and lead them to focus on rewards rather than on punishments (Anderson and Berdahl, 2002). Furthermore, power facilitates individuals’ movements toward targets that satisfy their personal goals (Keltner et al., 2003). Gruenfeld et al. (2008) demonstrated that power is associated with instrumental perception. In addition, possessing power activates goal-directed behaviors. For those with high levels of power, when compared to powerless people, the approaching of a social target is driven more by the target’s usefulness (defined in terms of the perceiver’s goals). This difference suggests that power promotes individuals to process social targets more as instruments than as people with personalities. That is, people with power approach objects based on their usefulness (goal-relevant objects).

Since the approach tendency activated by power decreases the perception of humanity and sexual objectification is a form of dehumanization (Heflick and Goldenberg, 2009), social power should promote sexual objectification. Indeed, Civile and Obhi (2016) studied Caucasian participants and suggested that power increased participants’ sexual objectification of sexualized members of the opposite gender but not those of the same gender, whereas the powerless did not show sexual objectification of either sexualized men or women. In addition, the authors found that this sexual objectification toward the opposite gender seen with Caucasian power holders occurred only when the targets were Caucasian but not Asian. They attributed these diverging results to the widespread media sexualization of Caucasian women but not Asian women, who are portrayed as housewives (Civile et al., 2016). These works conducted preliminary explorations of the associations between power and sexual objectification. However, it is unclear whether the effect of power on sexual objectification can be generalized to Eastern cultures, for example, Chinese culture. On the one hand, there are mixed findings about whether there is objectification in Eastern cultures. Some researchers argued that there is less objectification in Eastern cultures compared to Western cultures (e.g., Fredrickson and Roberts, 1997; Loughnan et al., 2015). Others found that participants from both Western (e.g., Belgium) and Eastern (e.g., Thailand) cultures regarded sexualized targets as having less competence and less agency than non-sexualized targets and administered more pain to sexualized targets (Wollast et al., 2018). This suggested that sexual objectification does exist in Asian cultures, although perhaps to a lesser degree than that of Western cultures. On the other hand, previous research did not take the particular social contexts (i.e., the postreform era) of China into consideration. There is no doubt that the 40-year opening-up and economic reform in China have witnessed a great improvement in the status of Chinese women. However, gender segregation and disparity are still present, if not increased, in the labor market (Ji et al., 2017). In the family sphere, it has been reported that there was a resurgence of Confucian patriarchal traditions (Ji, 2015). Pressures from the public labor market and the private family spheres encourage Chinese women to suffer more in both home and work than their Western counterparts, let alone in social power. Thus, it is of great significance to explore the effects of power on objectification under Chinese cultural contexts. Furthermore, the majority of previous studies have focused on behavioral outcomes, while the neural correlates underlying power effects on sexual objectification remain an open question.

## The Present Study

The present study was designed to establish whether the effects of social power on sexual objectification could be replicated in Chinese culture and to explore the underlying neural correlates. In Study 1, we conducted a behavioral study to replicate the effects of social power on sexual objectification in a Chinese sample. Given the high occurrence of sexual objectification and the prevalence of sexualized media all around the world, we hypothesized that sexual objectification exists in China as well and that power holders would process sexualized women more analytically, with more sexual objectification, than they would men.

Study 2 explored the possible neural correlates of the effects found in Study 1. We consider the N170 amplitude inversion effect to be a neural representation of configural body processing. Rossion et al. (2000) showed that inverted versus upright targets delayed and increased N170 when participants were recognizing faces, but this effect was not seen for inverted objects. Additionally, Stekelenburg and de Gelder (2004) suggested that larger N170 amplitudes and longer latencies occur for inverted rather than upright human bodies, which supports the configural processing of human body shapes. That is, human bodies might be processed configurally in the same manner as faces but not as objects. Minnebusch et al. (2009) also suggested that the N170 elicited by bodies is similar to the N170 elicited by faces. These studies demonstrated that the N170 component can be considered to be a robust indicator of configural body processing. Recently, researchers adopted the N170 amplitude inversion effect (i.e., larger N170 amplitudes for inverted vs. upright stimuli) to access cognitive objectification, with no N170 inversion effect indicating less configural processing and more cognitive objectification (Bernard et al., 2017, 2018a,c). That is, when presented with an inverted (vs. upright) stimulus, increased neural activity and cognitive resources are needed to detect the stimulus category, indicating greater configural processing (vs. analytical processing).

Similar to N170, P100 can also be affected by stimulus presentation (e.g., upright vs. inverted). Specifically, the P100 has a longer latency and larger amplitude in response to inverted than to upright faces (Itier and Taylor, 2004). However, body inversion does not affect P100 latency or amplitude significantly (Righart and de Gelder, 2007). Due to the functional dissociation between P100 and N170, researchers often use P100 as a baseline condition to examine the changes in N170 component (e.g., Minnebusch et al., 2009; Bauser and Suchan, 2013). We also included a P100 component in the present study and hypothesized that there would be an N170 amplitude inversion effect for power holders during the processing of sexualized men but not women. In addition, the pattern of P100 amplitude would not be same as that of N170; specifically, the amplitude inversion effect when processing both sexualized female and male stimuli would not occur on P100.

## Study 1

In Study 1, we intended to replicate the behavioral effects of power on sexual objectification. We hypothesized that participants in the high-power group would be more likely to process sexualized bodies analytically and there would be more objectification. Participants in the low-power and control groups would be more likely to process sexualized bodies configurally, that is, with less objectification. In addition, we predicted that there would be more sexual objectification of sexualized women than men.

### Methods

#### Participants

A total of 100 (57 women) undergraduate students participated in the study. They were recruited via an Internet advertisement on the BBS campus. All participants were Chinese and heterosexual by self-report on one question, “What is your sexual orientation?” The response choices were heterosexual or non-heterosexual (gay, lesbian, bisexual or other). All participants had normal or corrected to normal vision, and none had neurological disorders. Participants were randomly assigned to a high-power, low-power, or control group. There were 34 participants in the high-power group (19 women; *M*_*age*_ = 21.79 years, *SD* = 1.77), 36 in the low-power group (18 women; *M*_*age*_ = 21.42 years, *SD* = 1.81), and 30 in the control group (20 women; *M*_*age*_ = 21.17 years, *SD* = 1.72). We choose this sample according to the power manipulation of previous research (Galinsky et al., 2003; Hogeveen et al., 2013). Written informed consent was provided after procedural details had been explained. All procedures conformed to institutional ethical guidelines for research and were approved by the local ethics committee.

#### Procedures

##### Power manipulation

We manipulated social power by asking participants to recall a personal power episode. Participants were instructed to recall and write down a particular incident in their lives, including what had occurred and how they had felt in as much detail as possible, wherein they had possessed power over another individual or individuals (high-power group) or someone else had possessed power over them (low-power group; Galinsky et al., 2003), or to describe what had occurred to them on the previous day (control group; Hogeveen et al., 2013). The power recall task is very common and has been examined by previous researchers (e.g., Galinsky et al., 2003; Hogeveen et al., 2013; Civile and Obhi, 2016; Civile et al., 2016). We included a control group to examine the sexualized-body inversion effect reported in Bernard et al. (2012) and the power effects on objectification (Gruenfeld et al., 2008). All participants had 10 min to recall and write down the incident on a sheet of A4 paper with 21 lines.

##### Sexual objectification

After finishing the power-manipulation task, participants were instructed to complete an ostensibly unrelated image recognition task. This task, which was adopted from Bernard et al. (2012), was designed to measure sexual objectification. Participants were randomly presented with 48 images of sexualized males and females, who were in swimsuits or underwear, standing still, staring at the camera, and showing natural expressions. The stimuli images were also adopted from Bernard et al. (2012). There were 24 images of female (12 upright and 12 inverted) and 24 of male (12 upright and 12 inverted). To avoid the possible orientation tendency bias resulting from the stimulus images, we presented all images twice (one was the original, and the other was its left-right mirror image). As such, there were 96 trials in all (48 upright and 48 inverted). The experimental task was as follows: first, an image was presented for 250 ms, followed by a 1,000 ms blank screen. Next, participants were shown two images—the old image (presented previously) and its mirrored form—and were asked to identify which one was the image they had previously seen (the old image). They pressed the “*F*” key if the left one was the old image and the “*J*” key if the right one was the old image. There would be a new trial only after participants responded to the current images. We counterbalanced the positions (left vs. right) of the old images across trials. The image stimuli were presented in the center of a computer monitor using E-Prime 2.0 (Psychology Software Tools, United States). Participants’ recognition accuracy rates and reaction times were recorded. Subsequently, all participants were debriefed and compensated.

To avoid the potential effects of the familiarity and attractiveness of images, a pilot test with 17 participants (10 women; *M*_*age*_ = 22.88 years, *SD* = 1.80) was conducted. Participants were asked to rate the stimulus images in terms of familiarity (from 1 [*not familiar with it at all*] to 7 [*very familiar with it*]) and physical attractiveness (from 1 [*not at all*] to 7 [*very*]). For familiarity, the results suggested that there was no difference between male (*M* = 2.13, *SD* = 1.11) and female bodies [*M* = 2.23, *SD* = 1.05, *t*(16) = 0.78, *p* = 0.45, Cohen’s *d* = 0.10], indicating that participants were unfamiliar with both. For physical attractiveness, a 2 (stimulus gender: male, female) × 2 (participant gender: man, woman) repeated-measures analysis was conducted using SPSS version 16 (same as below). The main effect of stimulus gender was significant [*F*(1,15) = 7.61, *p* < 0.05, $\eta_{p}^{2}$ = 0.34], with female bodies (*M* = 3.82, *SD* = 0.94) being rated as more attractive than male bodies (*M* = 3.23, *SD* = 1.00). However, we did not find an effect of participant gender nor an interaction effect between stimulus gender and participant gender (all *p*s < 0.05). That is, participants tended to rate sexualized female bodies as more attractive than sexualized male bodies. According to previous research, the attractiveness of the target does not affect the performance of participants (Bernard et al., 2012, 2015, 2017; Civile and Obhi, 2016; Civile et al., 2016).

### Results

#### Manipulation Check

Two independent coders blind to the experimental groups rated the essays using a seven-point scale (1 = *no power at all*, 7 = *rich in power*) to determine how much power participants appeared to have exercised in the situation described (α = 0.74). As expected, the judges rated participants in the high-power group as significantly more powerful (*M* = 4.78, *SD* = 1.69) than those in the low-power group [*M* = 2.17, *SD* = 1.38, *t*(68) = 7.06, *p* < 0.001, Cohen’s *d* = 1.72] and marginally significant more power than those in the control group [*M* = 4.18, *SD* = 1.02, *t*(62) = 1.68, *p* = 0.10, Cohen’s *d* = 0.43]. Additionally, participants in the control group were rated as more powerful than those in the low-power group [*t*(64) = 6.62, *p* < 0.001, Cohen’s *d* = 1.66].

#### Recognition Performance *(d’)*

We conducted *d’* analysis with participants’ accuracy scores according to previous research (Stanislaw and Todorov, 1999). We computed the hit rates and false alarm rates of participants in each stimulus condition, computed the *d’* values for upright male bodies, inverted male bodies, upright female bodies and inverted female bodies, and used the *d’* values to conduct the following analyses.

A 2 × 2 × 3 × 2 mixed-model ANOVA was conducted, with stimulus orientation (upright, inverted) and stimulus gender (male, female) as within-subject factors, and power group (high, low, control) and participant gender (man, woman) as between-subject factors. Neither the four-way interaction nor the three-way interaction between participant gender, stimulus orientation and stimulus gender were significant (all *p*s < 0.23). However, an interaction effect of power group, stimulus orientation and stimulus gender was found [*F*(2,94) = 4.05, *p* = 0.02, $\eta_{p}^{2}$ = 0.08]. To dissect this interaction, separate 2 (stimulus orientation: upright, inverted) × 2 (stimulus gender: male, female) ANOVAs were conducted for each power group.

##### High-power group

**FIGURE 1:**
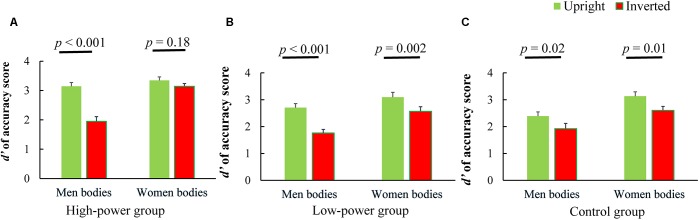
Results of the old/new recognition task in Study 1. **(A)** Participants’ performance in high-power group. **(B)** Participants’ performance in low-power group. **(C)** Participants’ performance in control group. Error bars indicate SEM.

We found a significant two-way interaction [*F*(1,33) = 25.90, *p* < 0.001, $\eta_{p}^{2}$ = 0.44]. There was a significant effect of stimulus orientation [*F*(1,33) = 38.65, *p* < 0.001, $\eta_{p}^{2}$ = 0.54] as well as stimulus gender [*F*(1,33) = 36.75, *p* < 0.001, $\eta_{p}^{2}$ = 0.53]. Simple effects analysis showed a significant inversion effect for sexualized males, whereby upright males (*M* = 3.14, *SD* = 0.75) were recognized better than inverted males [*M* = 1.95, *SD* = 0.95, *t*(33) = 7.92, *p* < 0.001, 95% CI = [0.89, 1.50], Cohen’s *d* = 1.41]; however, for sexualized females, participants’ recognition performance for upright (*M* = 3.35, *SD* = 0.67) and inverted bodies (*M* = 3.15, *SD* = 0.55) did not differ significantly [*t*(33) = 1.38, *p* = 0.18, 95% CI = [-0.10, 0.50], Cohen’s *d* = 0.33], suggesting that there was no body inversion effect (i.e., more objectification). We further calculated the Bayes factor of the null hypothesis (i.e., no significant difference between upright female bodies and inverted female bodies) against the alternative hypothesis (i.e., a significant difference between the two groups) using the Bayesian *t*-test (Rouder et al., 2009). The Bayes factor (*BF* = 2.30) supported the null hypothesis, suggesting that there was no inversion effect for female bodies. In other words, power holders processed sexualized males with less objectification and processed sexualized females with more objectification (see Figure 1A).

##### Low-power group

Again, an interaction between stimulus gender and stimulus orientation was found [*F*(1,35) = 5.68, *p* = 0.02, $\eta_{p}^{2}$ = 0.14]. Furthermore, the effects of stimulus orientation [*F*(1,35) = 46.24, *p* < 0.001, $\eta_{p}^{2}$ = 0.57] and stimulus gender [*F*(1,35) = 26.38, *p* < 0.001, $\eta_{p}^{2}$ = 0.43] were significant. Pairwise comparisons revealed a significant body inversion effect for sexualized males, with upright targets (*M* = 2.70, *SD* = 0.92) being recognized better than inverted targets [*M* = 1.76, *SD* = 0.79, *t*(35) = 7.77, *p* < 0.001, 95% CI = [0.70, 1.19], Cohen’s *d* = 1.11]. The same pattern emerged for sexualized female bodies, although attenuated; participants tended to recognize upright females (*M* = 3.09, *SD* = 1.10) better than inverted women [*M* = 2.57, *SD* = 1.01, *t*(35) = 3.35, *p* = 0.002, 95% CI = [0.21, 0.84], Cohen’s *d* = 0.50]. In other words, in the low-power group, sexualized males and females were both processed with less objectification, as demonstrated by the inversion effect (see Figure 1B).

##### Control group

There was no significant interaction between stimulus orientation and stimulus gender [*F*(1,29) = 0.04, *p* = 0.84, $\eta_{p}^{2}$ = 0.002], but there were significant main effects of stimulus orientation [*F*(1,29) = 16.58, *p* < 0.001, $\eta_{p}^{2}$ = 0.36] and stimulus gender [*F*(1,29) = 42.99, *p* < 0.001, $\eta_{p}^{2}$ = 0.60]. Pairwise comparisons suggested a significant body inversion effect for male bodies, with upright males (*M* = 2.39, *SD* = 0.85) being recognized better than inverted ones [*M* = 1.92, *SD* = 1.07, *t*(29) = 2.45, *p* = 0.02, 95% CI = [0.08, 0.86], Cohen’s *d* = 0.50]. The same pattern emerged for female bodies, with participants tending to recognize upright females (*M* = 3.13, *SD* = 0.89) better than inverted females [*M* = 2.60, *SD* = 0.82, *t*(29) = 2.74, *p* = 0.01, 95% CI = [0.14, 0.92], Cohen’s *d* = 0.63] (see Figure 1C).

#### Reaction Time

Similarly, a 2 (stimulus orientation: upright, inverted) × 2 (stimulus gender: male, female) × 3 (power group: high, low, control) × 2 (participant gender: man, woman) mixed-model ANOVA was performed. A significant four-way interaction was not found, and none of the three-way interactions were significant (all *p*s < 0.07). However, we found a significant interaction between stimulus orientation and stimulus gender [*F*(1,94) = 9.49, *p* = 0.003, $\eta_{p}^{2}$ = 0.09]. This finding was due to participants spending more time attempting to recognize upright male bodies (*M* = 1250.35 ms, *SD* = 557.82) than upright female bodies [*M* = 1168.96 ms, *SD* = 529.33, *t*(99) = 2.59, *p* = 0.01, 95% CI = [18.98, 143.80], Cohen’s *d* = 0.15]. Furthermore, participants also spent more time trying to recognize inverted male bodies (*M* = 1507.72 ms, *SD* = 730.92) than inverted female bodies [*M* = 1266.93 ms, *SD* = 568.52, *t*(99) = 6.99, *p* < 0.001, 95% CI = [172.42, 309.16], Cohen’s *d* = 0.37]. These results suggested that the higher accuracy rate for female targets was not due to longer reaction times. In other words, we excluded the possibility of a speed-accuracy tradeoff induced by the experimental design.

### Discussion

In Study 1, we found an inversion effect in the high-power group when recognizing sexualized male bodies but not female bodies, suggesting that there was less configural processing for sexualized female and, accordingly, more sexual objectification. This finding is consistent with previous findings that suggested that power facilitates objectification (Gruenfeld et al., 2008) and sexual objectification (Civile and Obhi, 2016; Civile et al., 2016). Specifically, compared to participants in the control and low-power groups, participants in the high-power group were more likely to sexually objectify female bodies.

Some experts may speculate about the relatively low attractiveness ratings of the targets. In the present study, we adopted sexualized images from Bernard et al. (2012). In fact, the ratings of attractiveness in Bernard et al. (2012)’s work also appeared to be quite low (seven-point scale for physical attractiveness; sexualized female, *M* = 3.70, *SD* = 0.31; sexualized male, *M* = 3.04, *SD* = 0.27). However, recent work by Bernard et al. (2018c) suggested that it is not revealing clothing *per se* that matters for cognitive objectification, but suggestive postures. Thus, we believe that the ratings of the attractiveness of the targets were less important in the present study.

There are mixed findings about the effects of participant gender. Civile and Obhi (2016) found an effect of gender for both Caucasian man and woman participants who possessed power when processing opposite gender targets. However, other researchers reported no moderating effect of participant gender (Bernard et al., 2012, 2017; Gervais et al., 2012). We did not find a role of participant gender in the present study, which is consistent with the latter reports. One possible explanation is that sexual objectification toward women does not reflect in-group biases (Bernard et al., 2012, 2015) but is shared by both men and women, although each group has different motivations; sexual attraction might be the primary motive of sexual objectification for men, while women may be motivated by social comparisons to distance themselves from sexually objectified females (Vaes et al., 2011). There is an alternative explanation from the perspective of sociology. It has been suggested that the separation of public and private spheres have facilitated inequalities at home and at work for Chinese women (vs. men). Moreover, research has suggested that even women themselves subscribe to traditional gender role expectations and accept the disadvantaged position (Ji et al., 2017). In this case, the experience of power in daily life may be rare for women. Thus, the effects of the power recalling task in the laboratory may be limited for women. In contrast, for the advantaged men group, rich social power experiences may increase their access to a sense of power and increased objectification of sexualized women.

The body inversion effect found in the control group for both sexualized females and sexualized males seems to be inconsistent with previous findings (Bernard et al., 2012; Civile and Obhi, 2016). However, it can be explained when the stimuli that were used and the preferential perceptual style of the Chinese are taken into consideration. It has been suggested that social contexts play a critical role in the perceptual process of Asians, while Westerners’ perceptual style tends to focus on the foreground objects (Miyamoto et al., 2006). In Chinese sociocultural context, individuals are regarded as inherently connected to and involved in social relationships. Furthermore, Chinese focus more on cooperation, humanity, and perspective taking, which place many more restraints on individuals’ behavior. In turn, this may lead to a more configural cognitive processing style among Chinese. In contrast, in Western cultural contexts, individuals pursue their own goals without being influenced much by contexts, which may lead to a more analytic cognitive processing style (Miyamoto, 2013). In the present study, we adopted sexualized Caucasian targets, which are plain and 2-dimensional images, without background contexts. Thus, it is possible that both the holistic perceptual style of Chinese and the stimuli *per se* contributed to the presence of inversion effect for both sexualized males and sexualized females in the control group.

## Study 2

In Study 2, we investigated whether and how temporary shifts in social power modulate the neural correlates of the processing of sexualized bodies. We predicted that there would be both behavioral sexualized-body inversion effects and N170 amplitude inversion effects in the low-power group for both male and female stimuli, as well as for male stimuli in high-power group, while both the behavioral and EEG inversion effects would disappear in the high-power group for female stimuli. In addition, we predicted that the P100 amplitude pattern would not be influenced by either the stimuli gender or the stimuli position.

There are two differences between Study 2 and Study 1. First, given that the primary focus was on the effects of power on sexual objectification, we did not include a control group in Study 2. Second, Asian stimulus images (with their hands placed by their sides) were adopted in an old-new task (Civile and Obhi, 2016) in Study 2 to avoid the potential effects of both racial differences and physical features such as asymmetry during the mirror-image discrimination task.

### Methods

#### Participants

A new sample of 36 undergraduate student participants (17 women; *M*_*age*_ = 21.28 years, *SD* = 1.37) was recruited. All participants were Chinese and heterosexual by self-report and had normal or corrected to normal vision, and none had neurological disorders. Written informed consent was provided after the procedural details had been explained. All procedures conformed to institutional ethical guidelines for research and were approved by the local ethics committee.

#### Procedures

##### Power manipulation

Participants were randomly assigned to a high-power (17, 9 women) or low-power (19, 8 women) group, and the power manipulation procedure was the same as in Study 1.

##### Sexual objectification

We collected 208 copyright-free images of sexualized females and males from the Internet. All targets were in a standing position and had their hands placed by their sides in order to reduce physical asymmetry, and each of targets was presented as upright or inverted. Targets were all young and attractive, predominately Asian (greater than 95%), wearing underwear or swimsuits, and showing a neutral facial expression. The image stimuli were presented on a black background in the center of a computer monitor. We standardized the sizes of images to 373 × 560 pixels and changed all backgrounds to white. The size of each stimulus was 11.3 × 17.0 cm at a viewing distance of approximately 80 cm, resulting in a visual angle of 6 × 8°. The procedure consisted of two blocks, counterbalanced between participants. Each block consisted of a study phase and a test phase. There were 52 trials in the study phase and 104 trials in the test phase in each block. In the study phase, we presented 104 sexualized images, one at a time, with 26 of each kind of stimulus. The experimental sequence was as follows: first, a fixation point was presented for 1,000 ms; then, a stimulus image was presented in the center of the screen for 3,000 ms. This process was repeated until all images had been presented. In the test phase, participants were presented with 208 images, one at a time, wherein half were old images that had been viewed in the study phase and the other half were new images. Participants were instructed to identify as quickly and accurately as possible whether the stimulus was old (by pressing the *F* key) or new (by pressing the *J* key).

A pilot test with 18 individuals (all self-reported heterosexual; 10 women; *M*_*age*_ = 23.28 years, *SD* = 1.71) revealed that for familiarity, there was no difference between male bodies (*M* = 1.73, *SD* = 0.80) and female bodies [*M* = 2.16, *SD* = 1.26, *t*(17) = -1.90, *p* = 0.07, Cohen’s *d* = 0.42], suggesting that participants were unfamiliar with both. For attractiveness, an interaction effect between participant gender and stimulus gender was found [*F*(1,16) = 11.23, *p* = 0.004, $\eta_{p}^{2}$ = 0.41]. Pairwise comparisons revealed that male participants rated female bodies (*M* = 4.67, *SD* = 0.81) as more attractive than male bodies [*M* = 2.96, *SD* = 0.92, *t*(7) = -6.06, *p* = 0.001, Cohen’s *d* = 2.03], while female participants rated male bodies (*M* = 3.74, *SD* = 1.25) and female bodies (*M* = 3.26, *SD* = 1.03) as the same level of attractiveness [*t*(9) = -2.04, *p* = 0.07, Cohen’s *d* = 0.43]. These results accompanied with further analysis suggested that the familiarity and attractiveness of targets did not affect the performance of participants.

During ERP recordings, participants were asked to refrain from moving as much as possible. To retain the effect of power, participants were instructed to remember the previously recalled power experience between the two blocks for 2 min by asking themselves, “What was it? How did it happen? How did you feel?” Subsequently, all participants were debriefed and compensated.

##### Electrophysiological recordings and analysis

While participants were performing the power manipulation task and the old/new recognition task, EEG recordings were obtained from 64 scalp sites according to the international 10-20 system (Brain Products, Germany). Two additional electrodes were placed below the left eye and on the outer canthi of the right eye to record vertical and horizontal eye movements. The left and right mastoids served as references (average mastoid reference), and a ground electrode was placed at a medial frontal location. Electrode impedances were maintained below 10 kΩ. The data were continuously digitized at 1,000 Hz/channel and filtered with a bandpass filter of 0.5–35 Hz. ERP averages were computed off-line. All ERP data were preprocessed using the Brain Vision Analyzer version 1.0 (Brain Products). Eye movement artifacts were excluded. Epochs of 700 ms for each electrode, including a 100 ms prestimulus baseline, were time-locked to the onset of the image stimulus and sorted by experimental group. All trials in which EEG voltages exceeded a threshold of ±80 μV were excluded from the averaging (Stekelenburg and de Gelder, 2004). Finally, on average, 51.51 trails per condition were considered for further analysis. Moreover, a repeated mixed model ANOVA suggested that the number of remaining trails was not significantly different among the analysis cells (all *p*s < 0.20)

It has been suggested that N170 is larger over the right hemisphere than the left (Bentin et al., 1996). According to previous research, we measured P100 and N170 responses at four occipitotemporal electrode sites over the left and right hemispheres (P7/P8, PO7/PO8; Kovács et al., 2006; Rossion and Jacques, 2008). The P100 peak was defined as the maximum value between 60 and 140 ms after the stimulus, and the N170 peak was defined as the minimum value between 140 and 200 ms. Amplitude maxima were obtained to calculate latencies.

### Results

The recognition performance (*d’* of accuracy scores and reaction times), peak latencies, and amplitudes of P100 and N170 were submitted to repeated-measures ANOVAs with stimulus orientation (upright, inverted), stimulus gender (male, female), and hemispheric lateralization (left: P7/PO7, right: P8/PO8) as within-subject factors; and power group (high-power, low-power) and participant gender (man, woman) as between-subject factors. Greenhouse–Geisser adjustments to the degrees of freedom were used when appropriate.

#### Behavioral Results

##### Manipulation check

Participants in the high-power group were rated as significantly more powerful (*M* = 4.24, *SD* = 1.05) than those in the low-power group [*M* = 2.29, *SD* = 0.89, *t*(34) = 6.04, *p* < 0.001, Cohen’s *d* = 2.07; α = 0.69].

##### Recognition performance (d’)

Neither a four-way interaction nor a three-way interaction was found (all *p*s < 0.14). We found effects of stimulus gender [*F*(1,32) = 26.72, *p* < 0.001, $\eta_{p}^{2}$ = 0.46] and stimulus orientation [*F*(1,32) = 93.58, *p* < 0.001, $\eta_{p}^{2}$ = 0.75]. These effects were due to female bodies (*M* = 1.66, *SE* = 0.09) being recognized better than male bodies (*M* = 1.33, *SE* = 0.10), and upright bodies (*M* = 1.86, *SE* = 0.11) being recognized better than inverted bodies (*M* = 1.13, *SE* = 0.08).

##### Small-scale meta-analysis

Although Study 1 and Study 2 did not have completely consistent results for the effect of power on sexual objectification, to combine the results obtained from these different studies and to increase the precision of the parameter estimates, we computed a small-scale meta-analysis with the meta package in R 3.4.1. We did not include the control group from Study 1 that did not receive a power manipulation.

To examine the effects of the power on sexual objectification, we computed a new variable, *D*:

$$D_{M}=(d{’}_{Mu}-d{’}_{Mi})$$

$$D_{Fe}=(d{’}_{Feu}-d{’}_{Fei})$$

*d’*_*Mu*_ and *d’*_*Mi*_ represent participants’ recognition performances for upright and inverted images of sexualized males, respectively, while *d’*_*Feu*_ and *d’*_*Fei*_ represent participants’ recognition performances for upright and inverted images of sexualized females, respectively.

We conducted meta-analyses for the two power groups separately. Because there was low heterogeneity between the different studies, we adopted a fixed-effect model. The analysis of the studies examining differences between the recognition of female targets and male targets in the high-power group (*N* = 51) suggested a significant overall effect, Hedges’ *g* = 0.82, *p* < 0.001, 95% CI = [0.41, 1.23]. In addition, there was low heterogeneity across the two studies, *Q*(1) = 3.89, *p* = 0.05. However, in the low-power group (*N* = 55), the analysis did not find a significant overall effect, Hedges’ *g* = 0.27, *p* = 0.17, 95% CI = [-0.11, 0.64], and the heterogeneity across the two studies was low, *Q*(1) = 2.68, *p* = 0.10.

These analyses suggested that there were significant differences for high-power participants when recognizing male and female targets. Specifically, there were body inversion effects and, in turn, less sexual objectification for male targets, while there were no body inversion effects and more sexual objectification for female targets. However, there were no significant differences when recognizing both female and male targets for participants in the low-power group, indicating the absence of sexual objectification.

#### Electrophysiological Results

**FIGURE 2:**
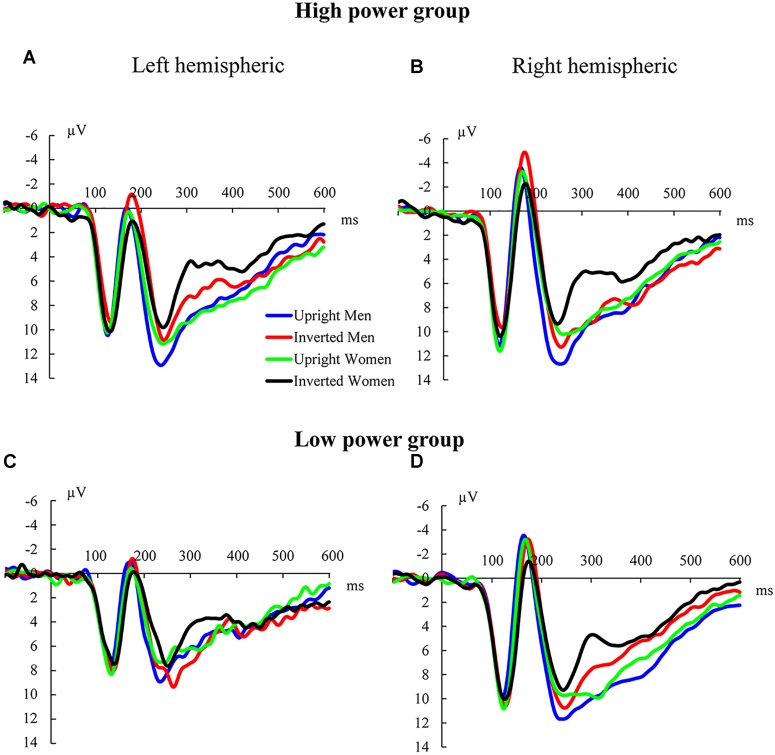
Grand average ERP waveforms for sexualized bodies. **(A)** Grand average ERP waveforms for the left hemisphere in the high-power group. **(B)** Grand average ERP waveforms for the right hemisphere in the high-power group. **(C)** Grand average ERP waveforms for the left hemisphere in the low-power group. **(D)** Grand average ERP waveforms for the right hemisphere in the low-power group.

We analyzed the EEG data from the test phase. Figure 2 shows the grand average ERP waveforms for the left and right hemispheres in response to upright male bodies, inverted male bodies, upright female bodies, and inverted female bodies for the two power groups.

##### N170

The repeated ANOVA on the N170 amplitudes revealed a main effect of hemispheric lateralization [*F*(1,32) = 17.81, *p* < 0.001, $\eta_{p}^{2}$ = 0.36], with larger N170 amplitudes for the right hemisphere (*M* = -4.52 μV, *SE* = 1.04) than the left hemisphere (*M* = -1.73 μV, *SE* = 0.95); there was also a main effect of stimulus gender [*F*(1,32) = 48.22, *p* < 0.001, $\eta_{p}^{2}$ = 0.60], with larger N170 amplitudes for male bodies (*M* = -3.76 μV, *SE* = 0.93) than female bodies (*M* = -2.49 μV, *SE* = 0.96). In addition, we found a marginally significant interaction between stimulus gender, stimulus orientation, and power group [*F*(1,32) = 3.88, *p* = 0.058, $\eta_{p}^{2}$ = 0.11].

Further analysis examined the two-way interaction between stimulus gender and stimulus orientation within the two power groups. We found a significant interaction effect in the high-power group [*F*(1,16) = 13.38, *p* = 0.002, $\eta_{p}^{2}$ = 0.46]. Pairwise comparisons revealed larger N170 amplitudes for inverted males (*M* = -4.50 μV, *SE* = 1.40) than upright males (*M* = -3.08 μV, *SE* = 1.30, *p* = 0.02, 95% CI = [0.25, 2.60]). In contrast, N170 amplitudes for inverted females (*M* = -2.13 μV, *SE* = 1.27) were similar to the N170 amplitudes associated with upright females (*M* = -2.89 μV, *SE* = 1.35, *p* = 0.19, 95% CI = [-1.92, 0.41]). The Bayes factor (*BF* = 1.80) supported the null hypothesis, suggesting that there was no N170 amplitude inversion effect for female bodies. Moreover, we did not find the predicted two-way interaction in the low-power group [*F*(1,18) = 1.31, *p* = 0.27, $\eta_{p}^{2}$ = 0.07].

A repeated measures ANOVA on N170 latencies revealed main effects of hemispheric lateralization [*F*(1,32) = 9.73, *p* = 0.004, $\eta_{p}^{2}$ = 0.23], stimulus orientation [*F*(1,32) = 74.60, *p* < 0.001, $\eta_{p}^{2}$ = 0.70], and stimulus gender [*F*(1,32) = 30.33, *p* < 0.001, $\eta_{p}^{2}$ = 0.49]. These effects were due to delayed N170 latencies for the left hemisphere (*M* = 173.71 ms, *SE* = 1.41) compared to the right hemisphere (*M* = 170.54 ms, *SE* = 1.23); delayed N170 latencies for female bodies (*M* = 174.02 ms, *SE* = 1.31) compared to male bodies (*M* = 170.23 ms, *SE* = 1.23); and delayed N170 latencies for inverted bodies (*M* = 176.68 ms, *SE* = 1.43) compared to upright bodies (*M* = 167.57 ms, *SE* = 1.22).

##### P100

A repeated measures ANOVA on P100 amplitudes showed main effects of hemispheric lateralization [*F*(1,32) = 11.38, *p* = 0.002, $\eta_{p}^{2}$ = 0.26] and stimulus gender [*F*(1,32) = 4.80, *p* = 0.04, $\eta_{p}^{2}$ = 0.13]. These effects were due to larger P100 amplitudes for the right hemisphere (*M* = 11.96 μV, *SE* = 0.94) compared to the left hemisphere (*M* = 10.25 μV, *SE* = 0.85) and larger P100 amplitudes for female bodies (*M* = 11.29 μV, *SE* = 0.87) compared to male bodies (*M* = 10.93 μV, *SE* = 0.86). Next, an interaction between stimulus gender and hemispheric lateralization was found [*F*(1,32) = 4.25, *p* = 0.047, $\eta_{p}^{2}$ = 0.12]. Pairwise comparisons revealed that in the left hemisphere, male (*M* = 9.93 μV, *SE* = 0.92) and female (*M* = 10.05 μV, *SE* = 0.93) bodies evoked similar P100 amplitudes, but for the right hemisphere, female bodies (*M* = 12.06 μV, *SE* = 1.02) evoked larger P100 amplitudes than did male bodies (*M* = 11.50 μV, *SE* = 1.01). Additionally, there was a significant interaction between stimulus gender, stimulus orientation, and power group [*F*(1,32) = 7.24, *p* = 0.01, $\eta_{p}^{2}$ = 0.18]. Further analysis did not find a significant interaction effect between stimulus gender and stimulus orientation in the high-power group [*F*(1,16) = 1.16, *p* = 0.30, $\eta_{p}^{2}$ = 0.07]. However, we did find an effect in the low-power group [*F*(1,18) = 7.12, *p* = 0.02, $\eta_{p}^{2}$ = 0.28]. This finding was due to similar P100 amplitudes for upright males (*M* = 9.91 μV, *SE* = 1.17) and inverted males (*M* = 10.61 μV, *SE* = 1.37, *p* = 0.22, 95% CI = [-0.44, 1.83]) but significantly larger P100 amplitudes for upright females (*M* = 10.76 μV, *SE* = 1.26) than for inverted females (*M* = 10.00 μV, *SE* = 1.31, *p* = 0.04, 95% CI = [0.05, 1.46]).

A repeated measures ANOVA on P100 latencies revealed a main effect of stimulus orientation [*F*(1,32) = 40.44, *p* < 0.001, $\eta_{p}^{2}$ = 0.56], with delayed P100 latencies for inverted bodies (*M* = 127.49 ms, *SE* = 1.43) compared to upright bodies (*M* = 123.03 ms, *SE* = 1.29). An interaction between stimulus gender and stimulus orientation was found [*F*(1,32) = 13.29, *p* = 0.001, $\eta_{p}^{2}$ = 0.29]. Pairwise comparisons revealed that inverted bodies evoked delayed P100 latencies compared to upright bodies for both male and female participants (all *p*s < 0.005). Moreover, there was a significant four-way interaction effect between hemispheric lateralization, power group, stimulus gender and stimulus orientation [*F*(1,32) = 4.85, *p* = 0.04, $\eta_{p}^{2}$ = 0.13]. Further analysis suggested that in the high-power group, a three-way interaction emerged [*F*(1,16) = 4.65, *p* = 0.047, $\eta_{p}^{2}$ = 0.23]. There was no significant effect for the left hemisphere (all *p*s < 0.05), but for the right hemisphere, a two-way interaction effect was found [*F*(1,16) = 10.57, *p* = 0.005, $\eta_{p}^{2}$ = 0.41]. Pairwise comparisons revealed that there were delayed P100 latencies for inverted male bodies (*M* = 126.18 ms, *SE* = 2.50) compared to upright male bodies (*M* = 119.71 ms, *SE* = 2.06, *p* = 0.001, 95% CI = [-9.79, -3.15]). In contrast, upright women (*M* = 123.38 ms, *SE* = 2.39) evoked similar P100 latencies as inverted women (*M* = 123.76 ms, *SE* = 2.99, *p* = 0.75, 95% CI = [-2.83, 2.06]). In the low-power group, we did not find a three-way interaction [*F*(1,18) = 2.03, *p* = 0.17, $\eta_{p}^{2}$ = 0.10]. Pairwise comparisons revealed that inverted bodies evoked larger P100 latencies than did upright bodies (all *p* < 0.05) regardless of stimulus gender.

### Discussion

Study 2 investigated the effects of power on sexual objectification using the body inversion effect and N170 amplitude inversion effect as indicators of configural processing. We did not find the body inversion effect on behavioral recognition performance. It is possible that the limited sample size of the ERP study contributed to the absence of power effects. An alternative explanation from Sturm and Antonakis (2015) is that the power manipulation matters. It is reported that although the power recall task is common for previous power research (e.g., Galinsky et al., 2003; Hogeveen et al., 2013; Civile and Obhi, 2016; Civile et al., 2016), there are possible demand effects; that is, participants recall their power experiences under the demand of the experimenter. The demand effects might confound the reality of the power from the power-related experiences. In addition, the manipulation check by two blinded coders cannot distinguish the power experienced in the recalled experience from the demand effects. The relatively lower power in the high-power group (*M* = 4.24, *SD* = 1.05) may indicate demand effects. However, the small-scale meta-analysis suggested a robust body-inversion effect in the high-power group, which suggested effects of power on objectification. In addition, the electrophysiological data suggested that power holders processed sexualized female bodies with more sexual objectification, as evidenced by the absence of N170 amplitude inversion effects, while N170 amplitude inversion effects were seen for male bodies, showing less sexual objectification. In contrast, powerless participants showed a significant N170 amplitude inversion effect for both sexualized male and female bodies. In addition, a right hemisphere-specific N170 inversion effect was found in the present study, which accounted for the right hemispheric effects of N170 amplitudes (Bentin et al., 1996).

Rather than clearly demarking stages, we consider these components (P100, N170) to represent a continuous and progressive accumulation of information about the visual stimulus encountered—in this case, a sexualized body. Larger N170 amplitude inversion was found when processing sexualized male bodies than when processing female bodies for power holders but not for the powerless. However, there were larger P100 amplitudes when processing upright females compared to processing inverted females but similar P100 amplitudes for upright males and inverted males for participants with low power. These results support previous studies that suggested that power increases the analytical processing of targets, leads to more objectification, and induces an N170 amplitude inversion effect. Having power grants individuals relative freedom from punishment and, in turn, leads to various perceptual cognitive effects. Power holders are more likely to perceive sexualized women as objects with reduced humanity (e.g., competence, morals, enthusiasm; Gruenfeld et al., 2008). The N170 is considered to be the earliest ERP response to represent facial features coding in the visual system (i.e., a face-selective component). In addition, as early-stage electrical activity, the N170 can be considered to be an indicator of automatic processes (Kubota and Ito, 2007). An N170 response suggests that the analytical processing of sexualized women is more automatic (Bernard et al., 2017), which could be related to the notion that objectifiers require considerable cognitive resources to resist objectification (Tyler et al., 2017).

Contrary to N170 amplitude results, we did not find an interaction between stimulus gender and stimulus orientation for N170 latency; this finding is consistent with previous research that suggested that the N170 latency is not a robust indicator of an inversion effect (Bernard et al., 2017), as it can be delayed by inverted bodies, intact bodies (Stekelenburg and de Gelder, 2004), and bodies with disrupted spatial relationships among body parts (Minnebusch et al., 2009; Bauser and Suchan, 2013). Another interesting result is that there were longer N170 latencies for inverted bodies (vs. upright bodies) and female bodies (vs. male bodies). The underlying reasons for this effect might be that the inversion disrupts the configural information of bodies and that there are more specific features to recognize on female bodies (e.g., hair style), which increases the difficulty in detecting and encoding a body template for inverted bodies and female bodies, which, in turn, leads to longer reaction times.

We observed enhanced P100 latencies when bodies were presented in an inverted orientation. This finding is inconsistent with a previous study that suggested body inversion did not affect P100 latency or amplitude significantly (Righart and de Gelder, 2007). The P100 component is considered to be the earliest face-sensitive component, and it occurs following a brief glance at a face. The inversion effect on the P100 may be due to low-level differences between upright and inverted faces (Jacques and Rossion, 2007). However, some studies have suggested that the P100 component is also sensitive to face orientation (Itier and Taylor, 2004). In this case, one possible explanation for the emergence of the P100 effect in the present study may be due to the whole-body stimulus we used. That is, the effect may reflect the presence of the faces but not the body parts of the stimuli. However, explanations must remain speculative at this stage. Another result worthy of attention is the presence of an interaction between stimulus gender and stimulus orientation for participants in high-power group in right hemisphere P100 latencies. The P100 latency inversion effect was significant for male bodies but not for female bodies in the high-power group, but this effect did not emerge in the left hemisphere or in the low-power group. On one hand, these findings could be due to the effects of power; on the other hand, research has suggested that P100 responses to an inversion effect may parallel the N170 responses, which are more pronounced in the right hemisphere than in the left (Bentin et al., 1996; Itier and Taylor, 2004).

## General Discussion

Across the two studies, we examined whether social power leads to increased sexual objectification using behavioral and ERP designs in Chinese samples. We found that being primed to social power increased participants’ sexual objectification toward sexualized women and that this effect was not affected by participant gender. The results support the large spread of sexual objectification in contemporary cultural practices (Fredrickson and Roberts, 1997; Bernard et al., 2017).

By extending prior research regarding power and sexual objectification, the current research contributed a great deal of information. First, this study is the first to explore the effects of social power on sexual objectification using a combination of behavioral and electrophysiological evidence. The current research revealed that the effects of power on the processing of sexualized bodies were seen not only by increased analytical processing with a lower body inversion effect (Civile and Obhi, 2016; Civile et al., 2016) but also by a lower N170 amplitude inversion effect. These findings suggest that when participants were primed with social power, approach-related processes were activated, which promoted analytic processing toward sexualized women.

Second, we extend the objectification theory by using Chinese samples and found that sexual objectification exists in Eastern culture, especially under power conditions. Sexual objectification has been suggested to be common in Western cultures (Fredrickson and Roberts, 1997; Bernard et al., 2012; Loughnan et al., 2015). However Loughnan et al. (2015) suggested that cultural norms could affect self-objectification and other-objectification and that there is more objectification in traditional nations of objectification research (e.g., Australia, Italy, United Kingdom, and United States) than in non-traditional nations (e.g., India, Japan, and Pakistan). We found that the inversion effect was restricted to sexualized women in Chinese cultural context, which is consistent with the initial finding in Bernard et al. (2012). However, recent studies by Cogoni et al. (2018) confirmed that the degree of sexualization and the visual properties of targets, as well as participants’ attention biases, all accounted for the presence/absence of inversion effect. In addition, the extent of sexualization of the targets affected the inversion effect for both male and female targets. Bernard et al. (2018c) also added evidence that the effects of postural suggestiveness on cognitive objectification were not moderated by target gender. However, previous research mainly focused on Caucasian participants, and no certainty exists on whether these patterns of results also apply to the Asian population, which makes our study important to fill this gap.

According to findings from sociology, the gender inequality in postreform urban China has increased greatly in the past 40 years. The socialism-to-market transition of the state facilitates the collapse of the *danwei system* (i.e., work-unit of the State-owned enterprise and collective-owned enterprise in the socialist era) and the decline of Marxist egalitarian ideology, which, in turn, promote the resurgence of Confucian patriarchal traditions. The changes in the public sphere and private sphere as well as the state gender ideology all contribute to the separation of the family and the labor market. Chinese women suffer much more family-work conflict than their male counterparts (Ji et al., 2017). Within this cultural context, the unfavorable situation of Chinese women compels them to struggle to find a balance between work and family. In this case, the objectification of women by men (i.e., the advantaged group) and women themselves (i.e., the disadvantaged group) could be more prevalent, as suggested by the saying, “Marrying well counts more than career.” Actually, it suggested that there is a predominant role of husbands’ class positions in determining married women’s happiness (Chen, 2018). Thus, it is possible that men are usually the powerful and the objectifier while women are the powerless and the objectified, even for those women participants primed with high power in the laboratory. On the other hand, the restriction of sexual expression in traditional cultures may lead to the reduced sexually objectified portrayal of women in Asian media compared to Western cultures (Civile et al., 2016). In summary, our findings provide evidence that sexual objectification is common to Western and Eastern cultural practices, especially in the power contexts. These results have implications for understanding those females in the workplace who may take sexual objectification for granted.

We should note some limitations of the current work, which, in turn, suggest potential avenues for future research. First, although previous research has suggested that the concepts and behavioral tendencies associated with power can be activated whenever the possession of power is involved, consciously or unconsciously (Galinsky et al., 2003), the lack of power situation cues that resulted from the power recalling task and the limited power experiences of college students might limit the generalizability of the findings. Thus, research including actual superiors and subordinates would allow for greater ecological validity. Second, although our findings extend the study of sexual objectification into Eastern cultural practices and found effects of power on sexual objectification within Chinese culture, we cannot answer the question of cross-cultural effects. Future research could investigate cultural differences in the effects of power on objectification in various cultural populations and take cultural variables into consideration. Third, we adopted body-inversion paradigm in behavioral experiment and old-new task in EEG experiment. There may be confounds due to the former being focused on immediate memory processing and the latter on early visual processing (140–200 ms; Bernard et al., 2018c). Future research would benefit from unifying the paradigms adopted, although there is evidence of cognitive objectification from early visual processing (Bernard et al., 2017, 2018a), attentional processing (Nummenmaa et al., 2012; Gervais et al., 2013; Bernard et al., 2017), immediate memory processing (e.g., Bernard et al., 2015; Civile and Obhi, 2016), and long-term memory processing (Gervais et al., 2012). In addition, the different asymmetry between upright and inverted images presented is a fundamental limitation in Study 1. Although Bernard et al. (2018c) found that posture suggestiveness in the recognition paradigm was more responsible for cognitive objectification, this does not necessarily imply that it has the same effect in our Study 1. Cognitive objectification should be present regardless of the kind of task adopted to measure it, but the mediating or moderating variables matter. Future studies are needed to consider and control the presence of those perceptual confounds, such as asymmetries, complexity, colors, and so on (Cogoni et al., 2018). Finally, it is reported that Asian women are usually portrayed in mass media (e.g., advertisement) in their traditional gender roles in family, such as housewives and mothers (Frith et al., 2005), while Asian men are portrayed as bread winners with mental and civil abilities (Yang et al., 2005). Thus, future investigation of sexual objectification will benefit from taking the specific portrayal of sexualized males and females into consideration.

In sum, across two studies, the present work provides both behavioral and neural evidence of the effects of social power on sexual objectification in a Chinese cultural context. These findings could contribute to the extension of objectification theory and shed a new light on the explorations of the mechanisms of the effects of power on sexual objectification.

## Ethics Statement

This study was approved by the Ethics Committee of Southwest University of China. The experiments were conducted in accordance with the Declaration of Helsinki. And all participants gave written informed consent after detailed explanation of the experiments. Also after the experiments, they were paid for their participation.

## Author Contributions

LX conceived, designed, and conducted the study, analyzed the data, and wrote the manuscript. BL, FW, and LZ helped a lot in the mending of the manuscript. All authors involved in the research process, discussed results, and commented on the manuscript.

## Conflict of Interest Statement

The authors declare that the research was conducted in the absence of any commercial or financial relationships that could be construed as a potential conflict of interest. The reviewer GB and handling Editor declared their shared affiliation at the time of review.
